# The Sickle-Cell Fiber Revisited

**DOI:** 10.3390/biom13030413

**Published:** 2023-02-22

**Authors:** Marilyn F. Bishop, Frank A. Ferrone

**Affiliations:** 1 Department of Physics, Virginia Commonwealth University, Richmond, VA 23284, USA; 2 Department of Physics, Drexel University, Philadelphia, PA 19104, USA

**Keywords:** double strand, electron microscopy, sickle cell polymer

## Abstract

Sickle cell disease is the consequence of a single point mutation on the surface of the β chains of the hemoglobin molecule leading to the formation of rigid polymers that disrupt circulation. It has long been established that the polymers are comprised of seven pairs of double strands that are twisted replicas of the double strands found in crystals. Here, we review several newer developments that elaborate on that simple model and provide deeper insights into the process.

## 1. Introduction

Hemoglobin is a tetrameric protein—a dimer of dimers, 2 α and 2 β chains—that transports oxygen efficiently in higher organisms. Its efficiency derives from several features. Oxygen binding is cooperative, meaning that the protein can effectively sense if it is in an oxygen-rich environment (where it should thus favor binding) or an oxygen-poor region (that would favor delivery). The cooperative binding entails a shift in packing of the subunits as oxygen is released. Moreover, the red cell contains hemoglobin at almost ¼ of maximum packing density, and yet manages to behave as a collection of hard spheres whose only interaction is their mutual exclusion, thus further facilitating transport. In fact, the viscosity of a collection of red cells is essentially that of the hemoglobin molecules irrespective of being dispersed into cells [1].

**Figure 1:**
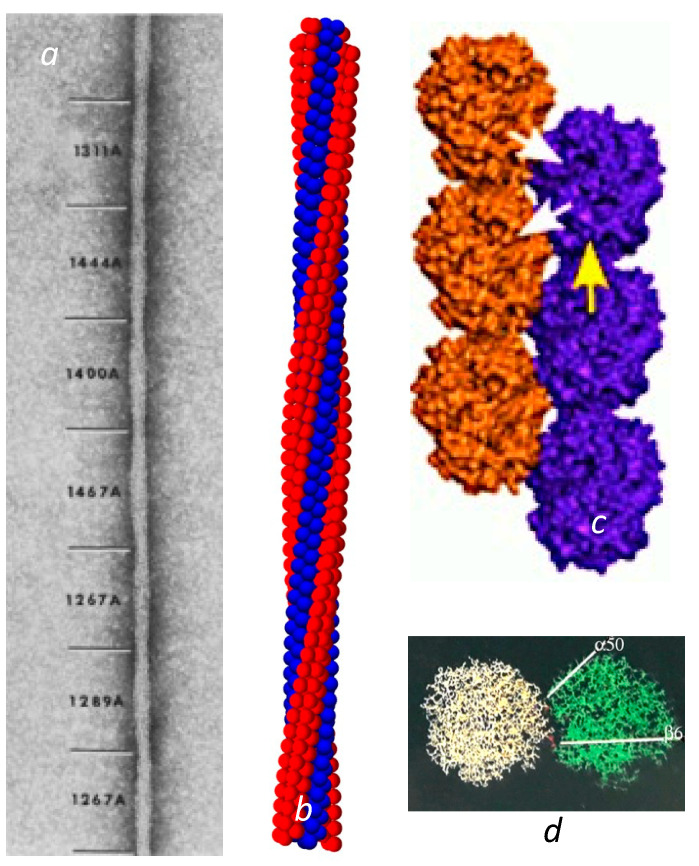
Fiber and double strands. (**a**) Electron micrograph of a negatively stained sickle fiber. Reprinted with permission from [7] 1988, Journal of Structural Biology. The undulating width is the consequence of the rotating elliptical cross section. The varying pitch is designated by the numbers shown in angstroms. (**b**) Reconstructed model of the fiber. The model is primarily based on Carragher et al. [8]. However, this is also the first demonstration that the meshing of four strands into a quad-strand [9] is fully compatible with a long 14-stranded fiber. (**c**) The crystal double strand [3]. Note that there are lateral contacts, shown with white arrows, where the mutation site is, as well as axial contacts as illustrated by the single yellow arrow. (For a detailed visualization of the location of all the amino acids in the contact region, the reader is referred to the figures in [3]). (**d**) The lateral interface viewed from the top (as down the axis of (**c**)). Not only does the β6 Val form a contact, but a distance away, α50 His also makes a contact that is markedly sensitive to pH.

Sickle cell disease is the consequence of a point mutation on the hemoglobin β chains that transforms Glu to Val on the molecular surface, and permits the deoxygenated Hb to assemble into long, multistranded fibers of roughly 20 nm in diameter. Given the intimate link between structure and function, an early and urgent goal for sickle cell research was to determine the structure of these fibers that rigidify the cells that contain them. State-of-the-art electron microscopy at that time was sufficient to reveal a twisted structure made of 14 strands [2] of undulating diameter, seen as the elliptical cross section rotated, with an average size of about 20 nm (Figure 1a). Unfortunately, electron microscopy was insufficient to determine the intermolecular contact sites.

Over time, a collection of sickle fibers will spontaneously transform into crystals, and in fact this is a method used to obtain HbS crystals for X-ray diffraction [3]. X-ray crystallography reveals that these crystals are composed of a set of double strands, in which each member of the pair is offset from its neighbor by half a hemoglobin molecule [3,4,5,6]. The mutation site Val β6 was found nestled in a hydrophobic pocket framed by Phe 85 and Leu 88 on another molecule’s β chain, located along the diagonal that connected the strands. This contact region is known as the lateral contact site. Along the individual strand axis is a second type of junction, known as the axial contacts. This region connects primarily β chains but is far more amorphous than the precise prongs of the lateral contacts (Figure 1c).

In the crystal double strand, a second important lateral contact is made between His α50, and Asp β79 at a point significantly separated from the primary lateral contact site of β6–β88 (Figure 1d). Having a second lateral contact point inhibits rotation of the HbS molecule around the Val β6 receptor pocket. This otherwise would be quite easy, as the Val fits somewhat loosely into the pocket wherein it is docked. At neutral pH, His α50 is only ionized about half the time [10]. When the His is uncharged, the ionic interaction with Asp β79 is lost, and the linear double strand becomes less favored. On this point, it is important to observe that the crystals are formed at pH 6.0 or lower, while the EM structures of 14-stranded polymers were all determined at pH 7 or above.

What happens, then, if electron microscopy (EM) is carried out on fibers formed at a pH closer to the crystal structure? With His α50 more tightly bound to its partner Aspβ79, new polymer structures appear, called macrofibers [11]. These are comprised of double strands and have far lower pitch than the 14-strand structure. Lacking a fixed number of double strands, macrofibers appear to have no set overall diameter, and generally are larger than 14-strand fibers.

These linear double strands that are known from crystalline form are considered to be integral elements of the twisted structure of the physiologically relevant 14-strand polymer. When the 14-stranded fiber was identified by EM [12] it was clear that the spacing and half stagger arrangement was the same as that of the crystal, leading to the hypothesis that the fiber was comprised of twisted double strands. Shortly thereafter, Edelstein [13] constructed a fiber based on the transformation of linear to twisted double strands and compared the results to previous tabulations of which mutants had effects on polymers. Josephs’ group at Chicago took this the furthest, building a structure that not only twisted the double strands, but further positioned them so as to optimize contacts that had been inferred from various biochemical studies [8,14,15]. Subsequent mutagenic studies have confirmed that the pockets found in the crystal lateral contacts, as well as the axial contacts, are functionally present in the fiber [16,17,18,19,20]. The lateral and axial intermolecular contacts are listed in Table 1 and Table 2.

With this paradigm in hand, it became possible to attempt to make a structural map of the fiber by twisting the double strands so as to map them onto the structure known from electron microscopy. Edelstein’s group and Josephs’ group both undertook this enterprise, and successively developed models of the fiber at atomic scale as a result. There is a degree of disagreement between these investigators about the density of packing of the fibers, with Cretegny and Edelstein [21] concerned that insufficient inter-strand contacts are formed in the Josephs structure, and Josephs et al. [15] contending that the map of Cretegny and Edelstein would involve molecular overlaps. Nonetheless, there is no dispute that the fiber is a composite of twisted double strands (Figure 1b). An aspect not considered by either is the potential for molecular disorder. Given that both groups were attempting to describe the structure that results from an average over many particle layers, it remains possible that the density issues are the consequence of inevitable imperfections within the structure. For example, even within the double strand, the molecules do not maintain a perfect reflection symmetry, a point made in the overview of these two viewpoints by Mu et al. [22].

In the hemoglobin tetramer, particularly when O_2_ or other ligands are bound to the iron of the heme, one αβ dimer readily dissociates from the other, subsequently recombining to retain the tetrameric structure. Mixtures of HbS and other Hb will therefore form hybrids. Such hybrids are an important feature of the pathophysiology of the disease. Sickle trait is the condition found in patients who inherit one S gene and one A gene, and thus carry a mix of Hb molecules in their red cells. Thanks to hybridization, a 50:50 mixture only carries 25% pure HbS, along with 25% HbA and 50% HbAS hybrids. This confers sufficient protection on such patients that their condition is essentially asymptomatic [23]. The drug hydroxyurea stimulates additional fetal hemoglobin (HbF) production [24], which likewise extends its influence by hybrid formation.

Since the double strand structure only used one β6 site, it followed that a hybrid would have a 50% chance of inserting into the polymer so as to make all the requisite contacts. Thus, the model of the polymer is commonly thought of as employing only half the contact sites that exist. Might the unused sites have any role in the pathophysiology of the disease? Slowly, important roles are emerging. While the story is not complete, our goal here is to provide an update on what is known and some insight into what may be on the horizon.

## 2. The Lateral Contacts and Heterogeneous Nucleation

**Figure 2:**
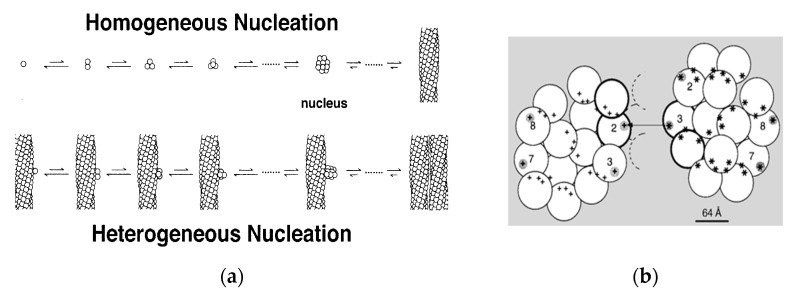
Kinetics and Mechanism. (**a**) The double nucleation mechanism [28]. Polymers can nucleate in homogeneous solution, or on the surface of pre-existing polymers. (**b**) The model of Mirchev and Ferrone [33]. Lateral contact sites on the polymer surface provide the template for heterogeneous nucleation by providing both donor and acceptor regions. This model has been experimentally verified [34].

Shortly after the 14-stranded fiber geometry had been deduced, a mechanism was also discovered that could explain the unusual assembly kinetics of sickle fibers. The rate of formation of the fibers was extraordinarily sensitive to solution conditions, such as concentration of HbS, with the early observations finding the reaction order to be about 30 to 40th power in concentration [25]. Yet the reaction strikingly showed an apparent latency, after which fibers explosively appeared. (The latency is called apparent because when highly sensitive probes were used, fibers were in fact discovered during the latent period [26]). The high concentration dependence suggested a nucleated assembly, but no known theories at the time could provide any explanation for the suddenness of the growth process once it commenced. In 1980, Ferrone et al. [27] proposed a novel mechanism that employed two nucleation steps: fibers that nucleated from solution in accord with well-known nucleation theories provided a template for a second nucleation process on their surface (Figure 2a). Because this was not in the homogeneous solution, but on a fiber surface, it was dubbed heterogeneous nucleation. The secondary path made the process exponential, while nucleation allowed for high concentration dependencies (augmented by molecular crowding). While a full treatment of this description was remarkably successful in describing the kinetics of assembly [28,29,30] and was validated by direct observation with DIC microscopy [31,32], the heterogeneous process was an empirical construct whose molecular origins were unknown when it was proposed. Indeed, the schematic drawings used to help visualize the mechanism (Figure 2a) show fibers growing side by side as the simplest to imagine, although at the time no actual structural information was available.

In 1997, Mirchev and Ferrone [33] showed that the amino acids that were present in the lateral contacts that were well known from the crystal and evident in the fiber structure also appeared on the surface of the fiber. Rudimentary model building immediately showed that these partners could meet if two fibers were appropriately docked, as seen in Figure 2b. Moreover, the angles made by the fibers were seen in certain electron micrographs [35]. Four of the fourteen molecules possess an external docking site. This is particularly important because the vast majority of fibers are formed via the heterogeneous nucleation pathway. The model predicted that fibers generated by secondary nucleation would grow at an angle, in contrast to the simple geometry of Figure 2a.

DIC microscopy that confirmed the double nucleation mechanism by visual observation showed fibers growing side by side. As shown by Mirchev and Ferrone, however, the ability of fibers to bend could permit fibers to launch at an angle and yet bend to a parallel geometry within the resolution of DIC methods. Briehl, Turner and coworkers [36] further have shown that not only does zippering of fibers occur but that it can be well characterized. Thus, what is known at present is consistent with the current structural model of heterogeneous nucleation.

The structural construct of Mirchev and Ferrone was tested in a rigorous way by constructing cross-linked hybrids of HbS and HbA, denoted HbASxl [34]. Such a molecule has only one β6 mutation site, and thus should be incapable of heterogeneous nucleation. When these hybrids polymerized exhibiting heterogeneous nucleation, it was first thought that the mechanism had been disproven. However, closer inspection revealed that the heterogeneous nucleation rate was, in fact, significantly suppressed, being some 4000 times less likely. The secondary nucleation that was thus observed in these experiments was attributed to the inevitable consequence of molecular disorder. The fact that heterogeneous nucleation was unlikely did not mean that it was impossible, since there would be a chance for a HbASxl hybrid to join the polymer by making an imperfect lateral contact. It must be noted that the lateral contact is not a single entity: a given molecule of HbS makes lateral contacts via donor and receptor regions. HbA lacks the donor Val β6, but still possesses the receptor pocket. Thus, the rare case of an HbASxl hybrid that had the αβ^A^ subunit in the polymer position would have the αβ^S^ subunit available for heterogeneous nucleation. The rarity of that occurrence is offset by the large number of sites on the surface of a fiber where it can occur.

## 3. Multiple Lateral Contacts

**Figure 3:**
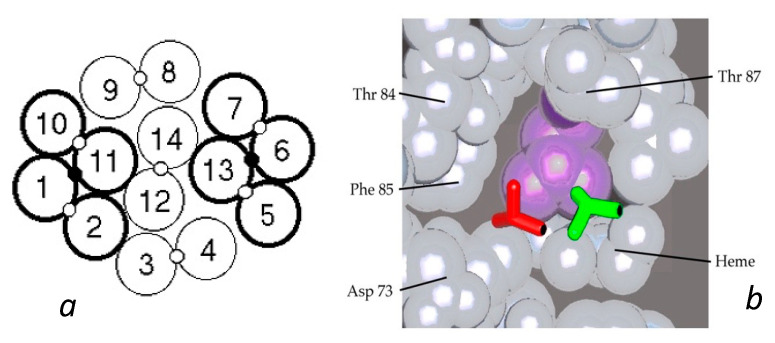
Interior new lateral contacts and polymer stability. (**a**) Cross section of the Carragher structure with the lateral contacts between the standard double strands shown as open circles between the 14 tetramers. By means of a small perturbation of that structure, an additional contact can be made as shown by the small black circle connecting strands 1 and 11, and 6 and 13 [9]. For convenience, such a cluster of two double strands is labelled a “quad strand”. (**b**) Comparison of the Val β6 shown as a licorice drawing docked into the acceptor pocket. The new contact (green) is only slightly displaced from that found in the X-ray structure of the double strands (red). The purple amino acid is Leu β88.

HbAS hybrids provided further important insights into the fiber structure. From the structure of the double strand, it was apparent that AS hybrids would have a 50% chance of entering the polymer, as described above. Yet data from several different laboratories consistently found the probability of being in the polymer to lie between 30 and 40%, with a single outlier [26,37]. Such an outcome is the natural result of having molecules within the fiber in which both lateral contacts were present (thereby allowing fewer hybrids to join the polymer). A proposal for this was put forth by Roufberg and Ferrone [9], who showed that a small perturbation of the position of two double strand pairs within the polymer structure could engage both β6 sites, effectively making new double strands within the fiber (see Figure 3). Rudimentary docking showed that there was little difference in the contact site geometry. While this was originally performed for a single layer of the fiber cross section, a complete fiber can indeed be constructed with this constraint as shown in Figure 1b. We also note that the second point in the lateral contact, the α50–β79 contact, is also made. With these added contacts, the probability of AS hybrids entering the polymer drops to 0.375 [9], a prediction in outstanding agreement with the data presently known.

The crosslinked AS hybrids described above provided a direct test of the notion that more than one lateral contact was the norm within the polymer. If only one lateral contact stabilizes the polymer, then AS hybrids would form a polymer with equivalent stability, measured by solubility, except for the small entropic term. The entropic effect comes from the fact that SS molecules would have two ways to join a polymer while AS would only have one. To avoid reproportionation of the subunits, they can be cross-linked.

And of course, cross linking SS molecules would provide a control experiment to ensure that the cross-link had not created new perturbations. The results confirm the use of more than one lateral contact. The solubility of HbASxl, first measured by Benesch et al. [38], was found to be 21.8 g/dL, as opposed to HbSSxl which was 13.5 g/dL. (The solubility of HbS without cross links was found by them to be 15.5 g/dL). Having only one of two ways to position each molecule within the polymer would not have increased solubility this much. Taking nonideality [26] into account, one would calculate an anticipated solubility of 17.6 g/dL for the cross-linked molecules, instead of the much higher 21.8 g/dL. That higher solubility is thus indicative of a weakened polymer due to the loss of the second lateral interaction. Similar solubility results were obtained by Rotter et al. [34], who also found the rates of nucleation were correspondingly slowed.

## 4. The Axial Contacts

Although the lateral contacts have been extensively studied by hybrid molecules and site-directed mutagenic approaches, the axial contacts are relatively unexplored. The axial contacts are far more diffuse and nonspecific than the lateral contacts, and of course the lateral contact is the site of the mutation that allows polymers to form. While the shape of the singular β6 mutation is intuitively visualized as a “donor”, and its pocket can be seen as “an acceptor”, the axial contact geometry does not enjoy such clarity. Nonetheless, one can somewhat arbitrarily label the region on one molecule a donor, and its complementary site as its acceptor, as illustrated by the white arrow in Figure 1c. Thus, each β chain has a donor and acceptor, with each tetramer therefore having the usual two donors and two acceptors. The axial contacts in the polymer are confirmed to contain amino acids found in the axial contacts of the double strand, again through mutagenic studies [19,20,39,40].

Perhaps most interesting is the Chiapas substitution of α 114 Pro → Arg in the axial contact region of HbS. Normal contact partners, highlighted in the axial contacts in Table 2, are Lys 16 and Glu 116, both also on α chains, as well as His 117, Phe 118 and Glu 121 on the β chains [19]. It is notable that α 114 makes contacts via both α chains. This means that a modification on either α_1_ or α_2_ 114 will also affect the polymerization in the opposite chain contact pairing and can provide some degree of inhibition. This is in contrast to situations described above where one chain is affected, and as a consequence there is a 50% copolymerization probability. Indeed, Ho et al. found 30% HbS-Chiapas mixed with HbS elevates solubility from 16.3 g/dL to 21.1 g/dL. HbS-Chiapas had a solubility above 33 g/dL, so could be considered essentially nonpolymerizing. The elevation of solubility as reported implies that HbS-Chiapas/HbS hybrids have only a 25% chance of entering the polymer [26]. We have recently carried out kinetic studies on this mutant, in which we find a substantial reduction in the nucleation rates, comparable to that seen in HbS/F mixtures. (Worth, Loll, Grasty, Fugate and Ferrone, in preparation).

## 5. Relative Strengths of the Contacts

An important quantitative issue is the relative strength of the axial vs. the lateral subunits. This is important because in the event of defects or imperfections in the polymer lattice, this will specify the relative populations of the types of intermolecular contacts likely to be lost. Wang and Ferrone [41] used light scattering to address this question by examining the amount of scattering from solution dimers below solubility as the temperature was raised. (In this context, dimers represent two full Hb molecules.) Since dimers are not favored, the free energy for their formation is positive, implying an unfavorable addition. What is significant, though, is that the species that do not polymerize, such as HbA or COHbS, are far less likely to form dimers than the HbS molecules. This difference corresponds to the difference in having the mutation site in the deoxy molecule, which is precisely the origin of the lateral contact. In contrast, the nonpolymerizing species have the option of forming an axial contact to generate a dimer, as well as any nonspecific other contact that is seen in the polymer. The difference between the free energy of dimer association is therefore a bound on the free energy difference between lateral and axial contacts. If there are more non-laterals, then the difference with respect to the axial contact is even greater. The outcome is that the lateral contact at least is some 2.2 kcal/mol stronger than the axial contact.

How strong is the contact energy? A relatively simple theory [28] for this process utilized in analyzing the nucleation rates suggests a chemical potential of about 7.5 kcal/mol is providing the attachment [42]. With two lateral contacts and two axial contacts, where the lateral is 2.2 kcal greater, one then has 0.8 kcal/mol in each axial and 3 kcal/mol in each lateral. An amount of 3 kcal/mol is also in close agreement with the cost of burying a hydrophobic surface of the size of the lateral contact [42]. What is interesting in the case of the axial contact is that this is not very far above kT (or RT), so that disruption of the axial contact is neither very costly nor terribly scarce. In addition, the prior discussion was based on the simple double strands. While a similar scaling should apply to quad strands, the details require simply too many assumptions to say much more than we have laid out above.

While the axial contacts maintain a geometry consistent with their name, it must be kept in mind that the lateral contacts are at about 45° with respect to the fiber axis, so that they play a role in the axial stiffness, for example, and any other behavior for which a vector component of that angled force is relevant.

## 6. Emerging Issues

With all that is now known about the geometry of the fiber and its constituent double strands, a point little addressed is why fibers twist. The typical model building studies have taken this as a given, and of course that is not an unreasonable starting point. However, the linear double strands seem to be the more stable form, yet nucleation sets the assembly on a course in which a twisted geometry must be ultimately undone. Based on preliminary studies, we believe there may be a simple but important answer to this question, based on the two points of contact in the axial regions, viz. the β6 and α50 amino acids. What we have found is that the optimum positions of these two points are in conflict. As the β6 becomes buried more completely, the salt bridge distance at α50 widens and thus weakens. However, when β6 is completely buried, the pairing generates a right-handed twist. To do this means that the strength of the salt bridge needs to be weakened just enough; thus, at a lower pH where the salt bridge strengthens, the strands begin to straighten out. It remains to be seen if this effect is more pronounced for small aggregates (i.e., nuclei).

## 7. Conclusions

At least three important structural issues still need further clarification. (1) Why do the fibers twist? The proposal mentioned above needs further investigation and testing. (2) What happens if the alternate axial contacts are used to build a polymer? The lateral contact alternatives provided much unexpected insight. Could the axial contacts do the same? (3) What role does disorder play? The low cost of error in the axial contacts suggests that erroneous packing, such as inverted molecules that keep a lateral contact but forego an axial one, could play a role in flexibility, rigidity and possibly the apparent variable pitch seen in Figure 1a. Elucidation of these points will continue to advance our understanding of the structure–function relationships that make this mutation a devastating disease, as well as providing insights into its cure.

## Figures and Tables

**Table 1:** Double strand lateral contact distances < 4.0 Å [3]. The two entries refer to the two non-equivalent molecules in the double strand, with the gray entries those that are greater than 4.0 Å. For the atomic details of the contacts, the reader is referred to the original work [3]. The contacts of the mutant Val β6 are highlighted.

	β_2_									α_2_	
β_1_	Thr4	Pro5	Val6	Ser9	Ala10	Ala13	Lys17	Pro125	Val126	Ser49	His50
Lys66		3.6 3.7									
Gly69		3.9 4.0									
Ala70		3.8 3.9	3.8 3.8								
Asp73	3.1 3.0		3.1 3.9								
Asn79										3.5 3.5	2.8 2.9
Asn80											3.7 3.7
Gly83								4.0 4.1		4.0 4.3	
Thr84			3.6 3.7								
Phe85			4.0 4.1								
Thr87					4.4 3.8	3.7 4.6			6.0 3.8		
Leu88				3.5 3.4							
Glu90							5.0 3.5				
Lys95							3.9 5.8				

**Table 2:** Double strand axial contact distances < 4.0 Å [3]. The two entries refer to the two non-equivalent molecules in the double strand, with the gray entries those that are greater than 4.0 Å. For the atomic details of the contacts, the reader is referred to the original work [3]. The contacts of Pro α114 are highlighted.

	α_2_					β_2_			
	Lys16	His20	Glu116	Pro114	Ala115	His116	His117	Phe118	Lys120
α_1_									
Pro114	3.4 5.9		3.7 3.9						
Ala115	4.8 3.7								
β_1_									
Gly16									3.1 3.5
Lys17						6.1 3.4	2.9 3.4	3.8 3.9	
Val18									3.2 2.9
Glu22		3.6 4.9							
His117				3.4 3.5	3.4 3.3				
Phe118				3.9 3.6	3.8 3.7				
Gly119					3.8 3.6				
Glu121				3.7 3.8					

## Data Availability

Not applicable.
